# Modulation of Tumorigenesis by Dietary Intervention Is Not Mediated by SIRT1 Catalytic Activity

**DOI:** 10.1371/journal.pone.0112406

**Published:** 2014-11-07

**Authors:** Katherine V. Clark-Knowles, Danielle Dewar-Darch, Karen E. Jardine, Michael W. McBurney

**Affiliations:** 1 Centre for Cancer Therapeutics, Ottawa Hospital Research Institute, Ottawa, Ontario, Canada; 2 Department of Medicine, University of Ottawa, Ottawa, Ontario, Canada; College of Tropical Agriculture and Human Resources, University of Hawaii, United States of America

## Abstract

The protein deacetylase SIRT1 is involved in the regulation of a large number of cellular processes that are thought to be required for cancer initiation and progression. Both SIRT1 activity and tumorigenesis can be influenced by dietary fat and polyphenolics. We set out to determine whether dietary modulations of tumorigenesis are mediated by SIRT1 catalytic functions. We introduced a mammary gland tumor-inducing transgene, MMTV-PyMT, into stocks of mice bearing a H355Y point mutation in the *Sirt1* gene that abolishes SIRT1 catalytic activity. Tumor latency was reduced in animals fed a high fat diet but this effect was not dependent on SIRT1 activity. Resveratrol had little effect on tumor formation except in animals heterozygous for the mutant *Sirt1* gene. We conclude that the effects of these dietary interventions on tumorigenesis are not mediated by modulation of SIRT1 catalytic activity.

## Introduction

SIRT1, the metazoan homolog of the yeast silent information regulator 2 (Sir2), is the most-studied protein of the seven-member family of mammalian protein deacetylases known as the sirtuins [1]. The sirtuins harbor a catalytic domain that couples NAD^+^-hydrolysis to protein deacetylation [2]–[4]. SIRT1 is a promiscuous enzyme, known to have a wide array of both histone and non-histone substrates [5]. Many of these substrates, such as PGC-1α [6], [7], p53 [1], [8], STAT3 [9], FOXO [10]–[12], and HIF1α [13] are implicated in the regulation of metabolic activity and modulation of these substrates by SIRT1 is thought to be critical for the ability of the organism to adapt to stress. Another subset of SIRT1 substrates are proteins with well-established roles in carcinogenesis. These include p53 [1], [8], p73 [14], RB [15], NF-κB [16], and c-MYC [17]. The overlap between these substrates is consistent with the idea that cellular metabolism and carcinogenesis are inextricably intertwined and that SIRT1 is a critical link in this interconnected network.

The rapid proliferation of cancer cells is associated with a metabolic shift from mitochondrial oxidative phosphorylation to aerobic glycolysis, a phenomenon known as the Warburg effect [18]. This process is dependent on the enzyme lactate dehydrogenase A, which produces NAD^+^ and lactate. The NAD^+^ produced in this manner may function as a cofactor for the SIRT1 enzyme [19]. If SIRT1, as a metabolic sensor, is a link between metabolism and carcinogenesis, then known modulators of SIRT1 activity could be exploited for preventative or therapeutic benefit. Identified modulators of SIRT1 activity include caloric restriction [20], a high fat diet (HFD) [21], [22], and a number of chemical compounds [23], [24] including the plant phytoalexin, resveratrol [25]–[27].

In mammals, caloric restriction (CR) can delay the onset of various ageing-related diseases including cancer [28]. CR has been associated with lifespan extension and in mice this phenomenon is thought to be dependent on SIRT1 activity [29]. This suggests that *Sirt1* may have the properties of a tumor suppressor gene and several studies have suggested that this may be the case [30]–[33]. Paradoxically, many studies suggest that *Sirt1* is an oncogene [8], [34]–[37]. Still other studies found that *Sirt1* has no effect on oncogenesis [38], [39]. These conflicting observations suggest that the role of SIRT1 in carcinogenesis is highly context-dependent, a view consistent with the notion that SIRT1 is a hub in a large scale-free network of proteins [5].

A relatively scant amount of clinical data has suggested an oncogenic role for SIRT1 in breast cancer [40]–[43], however many breast cancer cell lines have lost *Sirt1* alleles or contain mutations in the *Sirt1* gene, suggesting a role as a non-classical tumor suppressor [44]. Recently, we showed that the absence of SIRT1 catalytic activity neither suppressed nor promoted breast tumor formation and progression in mice carrying the MMTV-PyMT transgene [38]. These highly variable observations suggest that the molecular, physiological and environmental contexts are critical in defining the role of SIRT1 in cancer. We set out to determine whether dietary modulators of SIRT1 activity, high fat diet (HFD) and resveratrol, could influence tumorigenesis in mice with normal or mutant *Sirt1* genes. We report below that both dietary modifications modestly affected the onset of tumorigenesis but were not dependent on SIRT1 activity.

## Materials and Methods

### Animals

Male FVB/N-Tg(MMTV-PyMT)634Mul/J mice (hereto referred to as animals carrying the MMTV-PyMT transgene), were a generous gift from Dr. Bill Muller [45]. These male animals were crossed with female heterozygotes (*Sirt1* ^Y/+^), mice on a mixed 129sv/CD1 background and carrying the *Sirt1^tm2Mcby^* allele. This allele harbours a missense mutation in the *Sirt1* gene (H355Y) and encodes a catalytically inactive protein [38], [46]. Genotyping for the MMTV-PyMT transgene and the *Sirt1* alleles was performed as previously described [46], [47]. Mice were housed in groups of 2-4 animals, with a constant room temperature of 24°C and a 12 hour light/dark cycle.

### Diets

Animals received food and water ad libitum. Our previous work has shown that the caloric intake is similar between mice of all Sirt1 genotypes [48]. Specialty diets commenced at weaning. Mice in the high fat diet group received mouse diet containing 60% calories from fat (F1850 containing 3.1 kcal/gram, 36% fat, 20.5% protein, 36.2% carbohydrates, and 0% fiber; Bio-Serve, Frenchtown, NJ, USA). Standard diet contained 18% calories from fat (Teklad Global 2018 containing 5.51 kcal/gram, 6% fat, 18.6% protein, 44.2% carbohydrates, and 3.5% fiber; Harlan Laboratories). This diet study was carried out concurrently with our recently published study [38] and thus the standard diet control animal groups are the same animals as in that study. Resveratrol diet consisted of low phytoestrogen base chow (Teklad Global 2016 containing 3.0 kcal/gram, 4% fat, 16.4% protein, 66% carbohydrates, 3.3% fiber and up to 20 mg/kg phytoestrogen content, Harlan Laboratories, Madison, WI, USA) containing a final concentration of 417 mg of resveratrol (a generous gift from Biotivia, New York, NY, USA) per gram of chow. This concentration was calculated to achieve a final dose of 50 mg of resveratrol per kg of body weight per mouse per day, with control animals receiving low phytoestrogen base diet (Teklad Global 2016, Harlan Laboratories). We chose to use this dose as it is well within the range of doses where biological effects on metabolism attributed to resveratrol have been reported [49], [50]. Following weaning, animals were weighed weekly and digital palpation of the mammary glands was used to assess the presence of tumor. Mice were monitored until they had reached criteria for predetermined loss of wellness endpoint. These endpoints were defined as tumour burden where any tumour had a diameter of 20 mm, impaired mobility, tumour ulceration, and/or respiratory distress. All animal work described was carried out in accordance with *Guidelines for the Care and Use of Animals* established by the Canadian Council on Animal Care with protocols approved by the Animal Care Committee of the University of Ottawa, Ottawa, Ontario, Canada.

### Tissue Collection

Animals were euthanized via CO_2_ asphyxiation. Tumours were removed, weighed and fixed in 10% neutral buffered formalin. Lungs were examined for macroscopically visible metastatic nodules prior to fixation in formalin.

### Statistics

The probability of significant differences was determined by analysis of variance (ANOVA), employing the Kruskal-Wallis test with the Dunn's multiple comparison test. Survival and time-to-detection curves were compared using the LogRank test. Correlation was tested using the Spearman rank test. Data is expressed as mean±SEM (standard error of the mean) and *P*-values are two-sided. Analysis was performed using Graphpad Prism statistical software (Graphpad Software, San Diego, CA, USA).

## Results

### High fat diet decreased tumor latency but did not affect survival

Female mice carrying the MMTV-PyMT transgene develop mammary tumors that progress rapidly and metastasize to the lung [45]. We introduced the MMTV-PyMT transgene into stocks of mice carrying the H355Y point mutation [38] in the *Sirt1* gene (an allele referred to as *Sirt1* ^Y^ that encodes a protein with no detectable catalytic activity) and studied the emergence of mammary tumors in *Sirt1^+/+^*, *Sirt1^+/Y^*, and *Sirt1^Y/Y^* females. Animals were monitored weekly via digital palpation for the presence of any palpable mass in the mammary glands. It should be noted that no compensatory increase in the mRNA levels of other sirtuin family members, Sirt2-7, was observed in the Sirt1^Y/Y^ animals (Figure S1).

**Figure 1:**
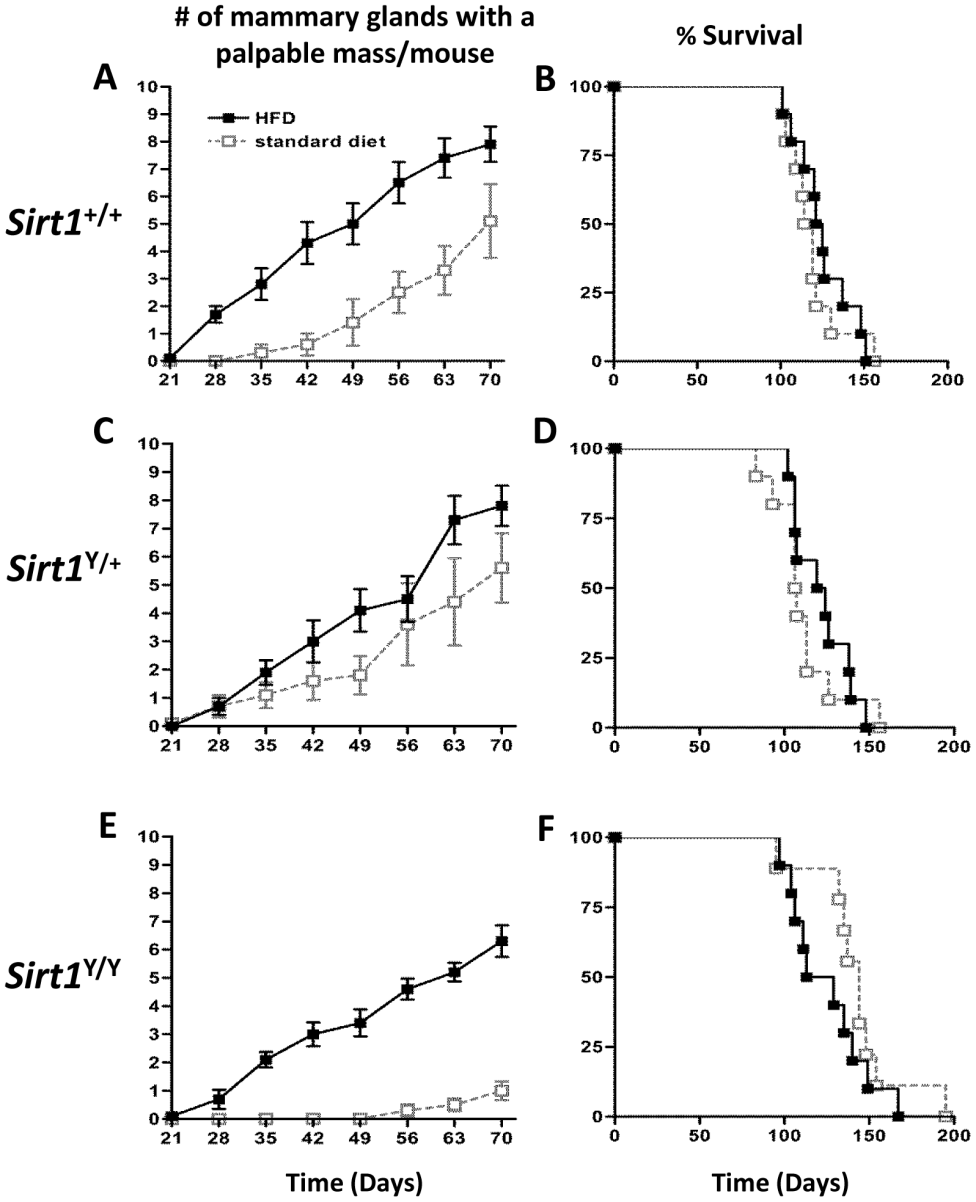
High fat diet decreased tumor latency but did not affect survival in MMTV-PyMT transgenic mice. **A, C, E)** Female mice of the indicated *Sirt1* genotypes carrying the MMTV-PyMT transgene were weaned onto high fat diet (HFD) or normal chow immediately following weaning. The mean number of mammary glands with a palpable mass was assessed at weekly intervals. Error bars indicate SEM. A significant difference in tumor latency was observed in *Sirt1* ^+/+^ (P<0.0001) and *Sirt1* ^Y/Y^ mice (P<0.0001). **B, D, F**) Kaplan Meier plot of surviving animals. N = 10 mice per *Sirt1* genotype per diet (□ standard diet,▪ HFD).

To investigate the effects of a high fat diet on tumorigenesis, mice were maintained on either a high fat diet (HFD) consisting of 60% calories from fat or a standard rodent diet (6% fat) from time of weaning until they had reached humane endpoint, generally due to significant tumor burden. HFD decreased tumor latency in both *Sirt1* ^+/+^ and *Sirt1* ^Y/Y^ mice (Figure 1A, 1E). The effect of HFD was particularly evident in *Sirt1* ^Y/Y^ mice that, at 70 days of age, had an average of 6 mammary glands with tumors while *Sirt1* ^Y/Y^ animals on standard diet had an average of only 1 gland with a tumor. However, the survival of neither *Sirt1* ^+/+^ nor *Sirt1* ^Y/Y^ mice on HFD was significantly reduced (Figure 1B, 1F). The HFD more modestly accelerated the onset of tumor detection in *Sirt1* ^Y/+^ mice (Figure 1C).

Prior to the commencement of the specialized diet, the *Sirt1* ^Y/Y^ mice weighed 20–30% less than their *Sirt1* ^Y/+^ and *Sirt1* ^+/+^ counterparts (Figure S2), which is typical for these mice [46]. On the standard diet, this difference was maintained over the course of the study. When placed on a HFD, the *Sirt1* ^Y/Y^ mice gained weight faster than their *Sirt1* ^+/+^ and *Sirt1* ^Y/+^ littermates. By 70 days of age (approximately 7 weeks on HFD) *Sirt1* ^Y/Y^ mice weighed an average of 10–20% more than the *Sirt1* ^+/+^ and *Sirt1* ^Y/+^ mice (Figure S2).

### High fat diet decreased metastasis to the lungs of *Sirt1^Y/Y^* mice

**Figure 2:**
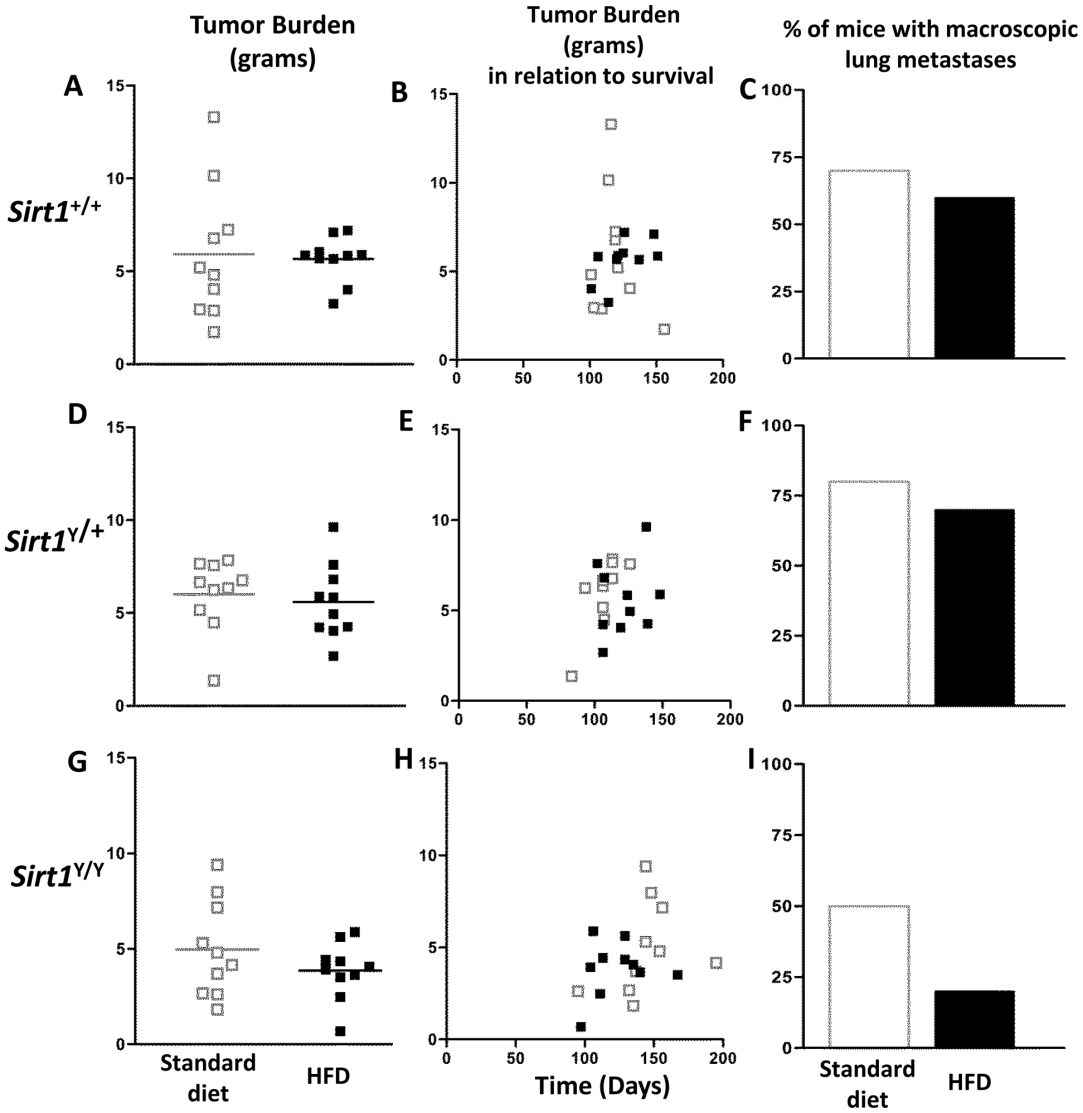
HFD had little effect on tumor burden. **A, D, G)** At experimental endpoint, animals were sacrificed and all mammary tumors were removed and weighed. Each point represents an individual animal. Mean tumor burden is represented by a line. No significant differences were evident. **B, E, H**) Tumor burden plotted against overall survival time showed no correlation. **C, F, I**) Percentage of mice with macroscopically visible metastatic lung nodules at necropsy. N = 10 mice per *Sirt1* genotype per diet (□ standard diet,▪ HFD).

Upon necropsy, all mammary tumors were removed and weighed. There was no difference in tumor burden between the two diet groups in *Sirt1* ^Y/Y^, *Sirt1* ^+/+^ or *Sirt1* ^Y/+^ mice (Figure 2A, D, G). There was no correlation between tumor burden and time of endpoint in the on either diet (Figure 2B, E, H).

Metastasis to the lungs is a frequent event in the etiology of mammary carcinomas that develop in the MMTV-PyMT mice. *Sirt1* ^Y/Y^ mice had fewer macroscopically visible metastatic nodules than *Sirt1^+/+^* or *Sirt1^+/Y^* animals on either HFD or normal chow (Figure 2C, 2F, 2I).

### Effect of resveratrol on tumor latency

**Figure 3:**
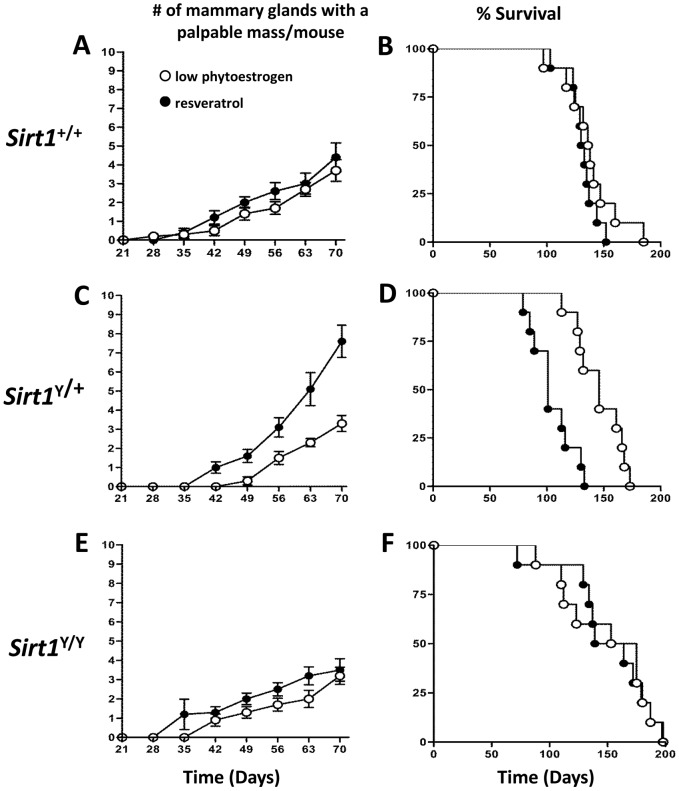
Resveratrol decreased tumor latency in *Sirt1^Y/+^* mice. **A, C, E)** The mean number of mammary glands with a palpable mass as assessed at weekly intervals following weaning. Error bars indicate SEM. In *Sirt1^Y/+^* mice, tumors appeared more rapidly in resveratrol treated animals (P<0.001). **B, D, F**) Kaplan Meier plot of surviving animals. *Sirt1^Y/+^* mice treated with resveratrol had reduced survival (P<0.001). N = 10 mice per *Sirt1* genotype per diet (○ low phytoestrogen control diet, • resveratrol diet).

Resveratrol has been reported to reduce the incidence of tumor formation and, in at least some cases, this effect is dependent on the SIRT1 protein [39]. To investigate the effect of resveratrol on tumors arising in animals carrying the MMTV-PyMT transgene, *Sirt1* ^+/+^, *Sirt1* ^Y/+^, and *Sirt1* ^Y/Y^ mice were fed a low phytoestrogen based diet supplemented with resveratrol. Treatment with resveratrol did not affect tumor latency in either *Sirt1* ^+/+^ or *Sirt1* ^Y/Y^ mice (Figure 3A, 3E) nor did resveratrol have an evident effect on survival duration (Figure 3B, 3F).

Unexpectedly, in the *Sirt1* ^Y/+^ mice, treatment with resveratrol decreased tumor latency as compared to the low phytoestrogen control diet (7.6±0.8 palpable masses by 70 days in resveratrol-treated mice versus 3.3±0.4 masses in mice in low phytoestrogen diet, P<0.001, Figure 3B). The resveratrol treatment resulted in reduced survival in these *Sirt1* ^Y/+^ animals with mean survival of 104±5.7 days vs 146±5 days for untreated mice (p<0.001, Figure 3D).

### Resveratrol had little impact on tumor burden or metastases

**Figure 4:**
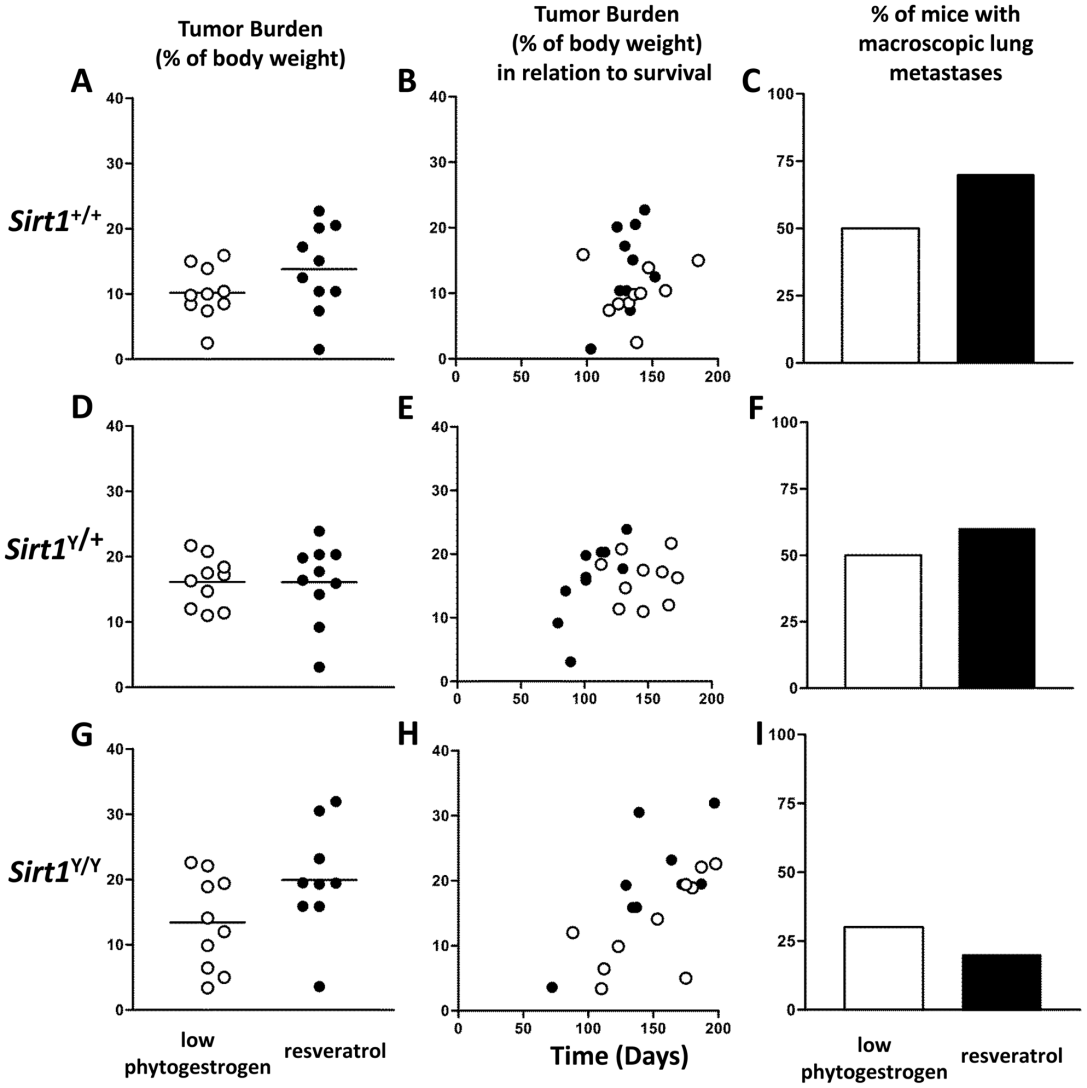
Resveratrol did not affect tumor burden. **A, D, G)** Each point represents the ratio of tumor burden to total body weight at endpoint. Mean percentage tumor burden is represented by a line. **B, E, H**) Tumor burden as a percentage of body weight at endpoint plotted against survival time. **C, F, I**) Percentage of mice with macroscopically visible metastatic lung nodules at necropsy. N = 10 mice per *Sirt1* genotype per diet (○ low phytoestrogen control diet, • resveratrol diet).

There was no significant difference in tumor burden for any of the *Sirt1* genotypes on either diet (Figure 4A, D, G). Tumor burden was weakly correlated with time of endpoint in *Sirt1* ^Y/Y^ animals on the resveratrol diet (r^2^ = 0.53) and animals on the low phytoestrogen control diet (r^2^ = 0.48) (Figure 4H).

*Sirt1* ^Y/Y^ mice were less likely to have macroscopically visible metastatic lung nodules than *Sirt1* ^+/+^ or *Sirt1* ^+/Y^ mice (Figure 4C, 4F, 4I). Resveratrol had only modest effects on the proportion of animals with lung metastases.

## Discussion

The role of SIRT1 in cancer is the subject of controversy [51]. The abundance or activity of SIRT1 has been reported to decrease or increase in tissues following treatment with HFD [22], [48] or resveratrol [52]–[54] respectively, so we set out to determine if the tumor promoting or inhibiting effects of these treatments on oncogenesis is dependent on SIRT1. The two treatments had bigger effects on tumorigenesis in mice with compromised SIRT1 activity. The parsimonious inference from these results is that neither HFD nor resveratrol affect tumorigenesis by modulating SIRT1 function. This conclusion is consistent with our previous work showing that development of skin tumors [39], intestinal tumors [39], and mammary tumors [38] in mice is not influenced by SIRT1 catalytic activity.

Obesity increases the risk of development and foreshortens survival in numerous cancer types, including breast cancer [55]. A diet high in fat is known to accelerate tumor development in mice carrying the MMTV-PyMT transgene [56], [57]. The SIRT1 deacetylase protects mice against the symptoms of metabolic syndrome resulting from a high fat diet [48], [58]. We found that the HFD significantly decreased tumor latency and this difference was greatest in *Sirt1* ^Y/Y^ animals. These *Sirt1* ^Y/Y^ mice also developed more severe obesity and symptoms of metabolic syndrome [48] consistent with the idea that factors associated with obesity accelerate tumor formation.

Resveratrol is a polyphenolic phytoestrogen found in grapes, peanuts, berries, and many other plant species, and dietary supplementation with this compound has become relatively common [59]. We previously showed that locally applied resveratrol protects mice from the development of skin papillomas and that this protection is largely dependent on SIRT1 [39]. Our study here showed that dietary resveratrol (sufficient to give a daily dose of 50 mg/kg) had little effect on the development of mammary tumors in either *Sirt1* ^+/+^ or *Sirt1* ^Y/Y^ mice. Curiously, resveratrol significantly accelerated tumor growth and decreased survival of *Sirt1* ^Y/+^ mice. Possible reasons for this are discussed below.

Mammary carcinogenesis is dependent on estrogen in the MMTV-PyMT transgenic mice [60], [61] and resveratrol has estrogenic properties in mammary tumor cells [62]. Indeed, mammary tumor promotion by resveratrol has been reported [63]. SIRT1 plays a modulating role in signalling by the estrogen receptor, with evidence pointing to the possibility of both a suppressive or enhancing effect [64]–[66]. The effects of estrogen receptor activation are dependent on the dosage of its cognate gene [67] suggesting that the tumor promotion seen with resveratrol in *Sirt1* ^Y/+^ animals may be a result of SIRT1 dosage-dependent modulation of estrogen receptor signalling. An analogous dose-dependent effect of SIRT1 on UV-induced skin carcinogenesis has been reported [68]. Conversely, this result may point to SIRT1-independent effects of resveratrol as have been reported [26], [69], [70]. Resveratrol has a number of established cellular target proteins such as the mitochondrial ATP synthase [71], quinone reductase 2 [72], and the estrogen receptor [73].

In all of the studies described here as well as in those reported previously [38] the *Sirt1* ^Y/Y^ mice developed fewer lung metastases than their *Sirt1* ^+/+^ and *Sirt1* ^Y/+^ littermates. Whether this due to an inherent property of the tumor cells or a consequence of a more hostile environment of the *Sirt1* ^Y/Y^ host tissue is not yet clear. Previous studies have shown that modulating tissue NAD^+^ concentrations can influence the metastatic properties of breast cancer cells [74] but whether this is mediated by SIRT1 (NAD^+^ is the co-substrate for SIRT1 catalysis) has yet to be investigated.

## Supporting Information

Figure S1 **There is no compensatory increase in expression of other sirtuin family members in tissues of Sirt1^Y/Y^ mice.** Sirt1-7 mRNA levels measured in **A**) liver (n = 1 per group) or **B**) brain (n = 2 per group) of Sirt1^+/+^ and Sirt1^Y/Y^ mice. Values are expressed as the mean ± SEM of the threshold cycle (C_T_).
(TIF)

Figure S2 **Weight gain over time.** *Sirt1* ^+/+^, *Sirt1* ^Y/+^, and *Sirt1* ^Y/Y^ (all carrying the PyMT transgene) mice were weighed once weekly and the weights represented here range from one week after commencing the specified diet (approximately 28 days of age) until the final week at which all animals in each group were still alive (70 days of age). **A**) standard rodent diet. **B**) high fat diet. **C**) low phytoestrogen diet. **D**) resveratrol containing diet. N = 10 for all groups.
(TIF)

File S1 **Materials and Methods: Quantitative Polymerase Chain Reaction (QPCR).** Measurement of Sirt1 mRNA levels via QPCR.
(DOCX)
