# Supplementary figures and images for: Modulation of Tumorigenesis by Dietary Intervention Is Not Mediated by SIRT1 Catalytic Activity
Source: PLoS One. 2014 Nov 7;9(11):e112406. doi: 10.1371/journal.pone.0112406 (PMC4224430; doi:10.1371/journal.pone.0112406)

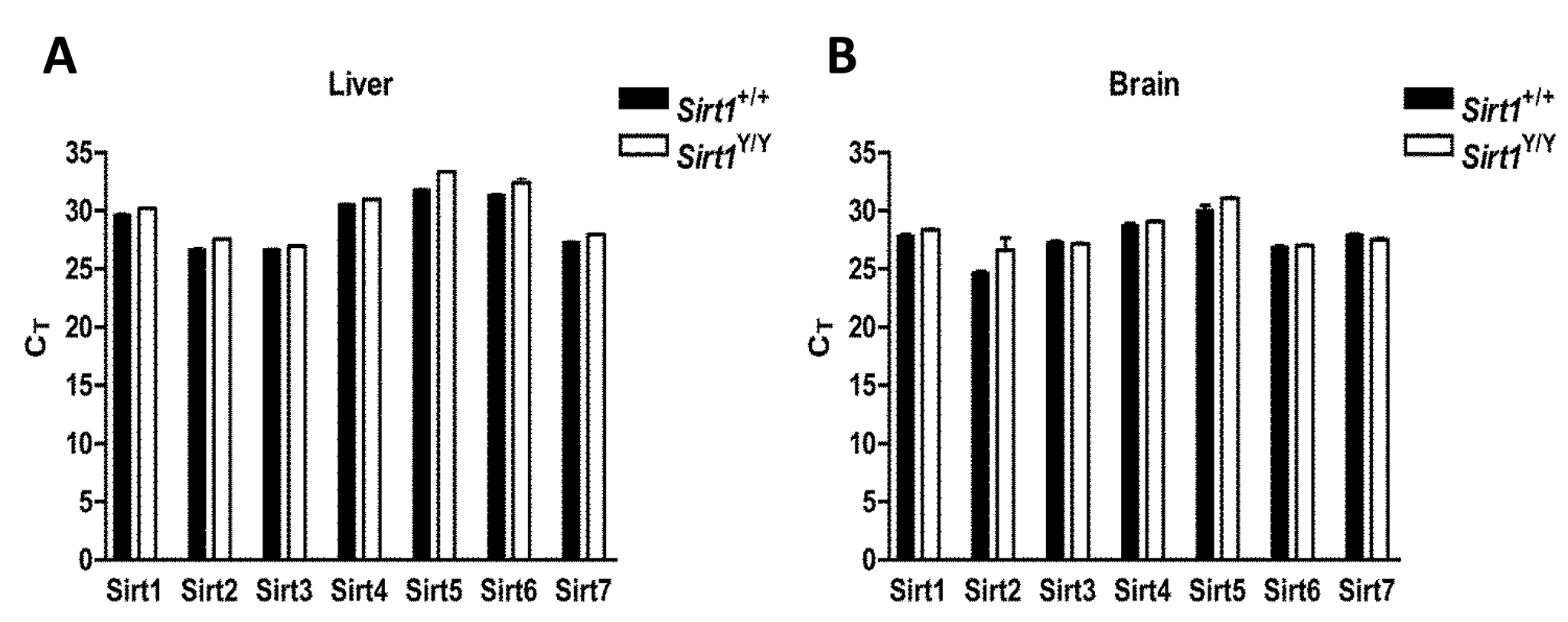

Supplement: Figure S1 — There is no compensatory increase in expression of other sirtuin family members in tissues of Sirt1Y/Y mice. Sirt1-7 mRNA levels measured in A) liver (n = 1 per group) or B) brain (n = 2 per group) of Sirt1+/+ and Sirt1Y/Y mice. Values are expressed as the mean ± SEM of the threshold cycle (CT). (TIF) [file pone.0112406.s001.tif]

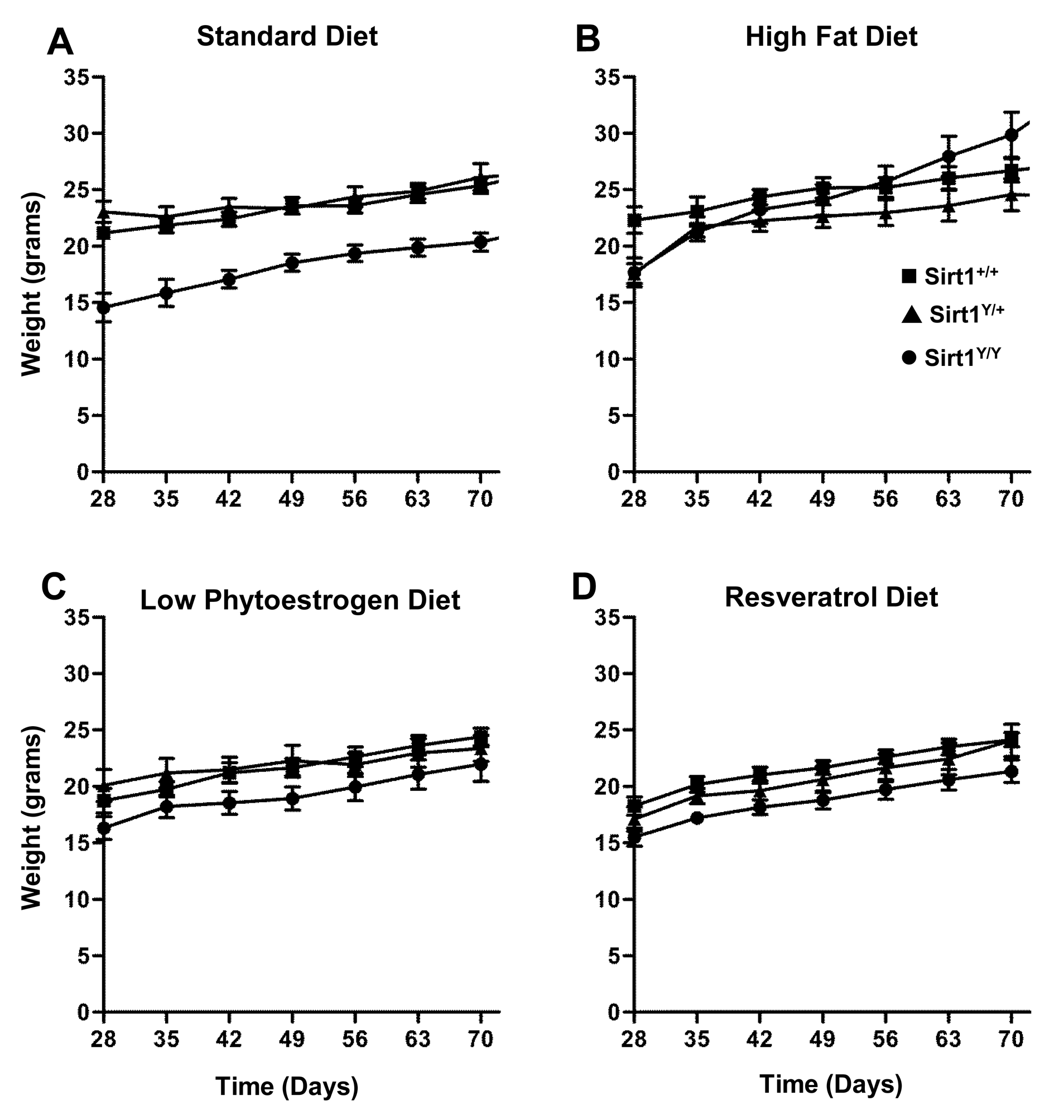

Supplement: Figure S2 — Weight gain over time. Sirt1 +/+, Sirt1 Y/+, and Sirt1 Y/Y (all carrying the PyMT transgene) mice were weighed once weekly and the weights represented here range from one week after commencing the specified diet (approximately 28 days of age) until the final week at which all animals in each group were still alive (70 days of age). A) standard rodent diet. B) high fat diet. C) low phytoestrogen diet. D) resveratrol containing diet. N = 10 for all groups. (TIF) [file pone.0112406.s002.tif]
