# Supplementary material for: Modulation of Tumorigenesis by Dietary Intervention Is Not Mediated by SIRT1 Catalytic Activity
Source: PLoS One. 2014 Nov 7;9(11):e112406. doi: 10.1371/journal.pone.0112406 (PMC4224430; doi:10.1371/journal.pone.0112406)
Supplement: File S1 — Materials and Methods: Quantitative Polymerase Chain Reaction (QPCR). Measurement of Sirt1 mRNA levels via QPCR. (DOCX) [file pone.0112406.s003.docx]

**Supporting File S1: Materials and Methods**

**Quantitative Polymerase Chain Reaction** Crude RNA was isolated from liver and brain of *Sirt1^+/+^* and *Sirt1^Y/Y^* mice using Trizol (Life Technologies, Burlington, Ontario, Canada) and then purified with the GenElute Mammalian Total RNA Miniprep Kit (Sigma, Oakville, Ontario, Canada). Random primed cDNA was transcribed from1 μg of RNA using the High Capacity cDNA Reverse Transcription Kit (ABI, Burlington, Ontario, Canada). cDNA samples were diluted 50X in Rnase/DNase free water and added to duplicate singleplex assays. Briefly, 5 μL of diluted cDNA was mixed with Taqman Fast Universal PCR Master Mix along with one of the following Taqman Gene Expression Assays (Life Technologies): *Sirt1* (Mm00490758_m1), *Sirt2* (Mm01149204_m1), *Sirt3*(Mm00452131_m1), *Sirt4* (Mm01201915_m1), *Sirt5*(Mm01351576_m1), *Sirt6*(Mm01149042_m1), and *Sirt7* (Mm01248607_m1). 18S (Hs99999901_s1) was used as an endogenous control. Reactions were analysed on an ABI 7500Fast expression system (ABI).
